# The Influenza A Virus H3N2 Triggers the Hypersusceptibility of Airway Inflammatory Response via Activating the lncRNA TUG1/miR-145-5p/NF-κB Pathway in COPD

**DOI:** 10.3389/fphar.2021.604590

**Published:** 2021-02-22

**Authors:** You-Hui Tu, Yan Guo, Shuang Ji, Ji-Long Shen, Guang-He Fei

**Affiliations:** ^1^ Department of Respiratory and Critical Care Medicine, The First Affiliated Hospital of Anhui Medical University, Hefei, China; ^2^ Key Laboratory of Respiratory Disease Research and Medical Transformation of Anhui Province, The First Affiliated Hospital of Anhui Medical University, Hefei, China; ^3^ Department of Pathogen Biology and Provincial Laboratories of Pathogen Biology and Zoonoses, Anhui Medical University, Hefei, China

**Keywords:** COPD, influenza A virus, airway inflammatory hypersusceptibility, lncRNA TUG1, miR-145-5p, NF-κB

## Abstract

**Background: **Patients with chronic obstructive pulmonary disease (COPD) are more susceptible to influenza A virus (IAV) with more severe symptoms, yet the underlying molecular mechanisms of the hypersusceptibility of airway inflammatory response remain unclear.

**Methods: **The primary human bronchial epithelial cells (pHBECs) were isolated from normal and COPD bronchial tissues (NHBE and DHBE) and cultured with/without IAV infection *in vitro*. DHBE cells were exposed to IAV for 24 h after knockdown of lncRNA TUG1 with short hairpin RNA (shRNA). Gain-of-function assays were performed with the miR-145-5p inhibitor and NF-κBp65 transfection. The expressions of lncRNA TUG1, miR-145-5p, phospho-NF-κBp65, NF-κBp65, TNF-α, and (Interleukin) IL-1β were examined with qRT-PCR, Western blotting, and ELISA. The interactions of lncRNA TUG1, miR-145-5p, and NF-κB were verified with luciferase reporter assay.

**Results: **The expressions of lncRNA TUG1, phospho-NF-κBp65, TNF-α, and IL-1β were increased significantly in pHBECs after being infected with IAV for 24 h (all *p*<0.05). The detailed time analysis revealed that the NF-κBp65 in DHBE was activated earlier than that in NHBE by Western blotting and immunofluorescence. Knockdown of lncRNA TUG1 and miR-145-5p mimic attenuated the expressions of NF-κBp65, TNF-α, and IL-1β significantly. The miR-145-5p inhibitor and NF-κBp65 transfection reversed the attenuated expressions of NF-κBp65, TNF-α, and IL-1β.

**Conclusion: **The IAV causes the hypersusceptibility of airway inflammatory response, which may be closely associated with more severe symptoms in AECOPD patients. The lncRNA TUG1 inhibitor may be a promising therapeutic strategy for AECOPD caused by IAV.

## Introduction

Chronic obstructive pulmonary disease (COPD) is a chronic airway inflammatory disease characterized by progressive airway inflammation and impaired lung function. Exacerbation of COPD (AECOPD) is the main source of hospitalization and mortality in COPD patients (Vogelmeier et al., 2017). The majority of exacerbation is related to respiratory viral infection (Wedzicha et al., 2014; Zwaans et al., 2014); accumulative evidence shows that influenza virus is one of the most frequently detected pathogens that induce AECOPD in Asian countries (Dai et al., 2015; Koul et al., 2017). Exacerbations triggered by viral infections are usually associated with hypersusceptibility of greater airway inflammatory response, more severe symptoms, and delayed recovery compared to those without viral infections (Almansa et al., 2012; Dickson et al., 2013). However, the underlying molecular mechanisms of the hypersusceptibility of airway inflammatory response induced by influenza A virus (IAV) in COPD patients remain unclear.

Long noncoding RNAs (lncRNAs) are defined as noncoding RNAs that have a length greater than 200 nucleotides, which may participate in the gene expressions of many inflammatory diseases, including COPD (Shi et al., 2013; Tang et al., 2016). The lncRNA taurine-upregulated gene 1 (TUG1) is a highly conserved lncRNA, originally identified in taurine-treated retinal cells. Previous studies have revealed the critical roles of lncRNA TUG1 played in the progression of various diseases (Li et al., 2016; Wang et al., 2019a; Su et al., 2020). Gu and colleagues found a higher expression of lncRNA TUG1 in the sputum and lung tissues of COPD patients as compared with nonsmokers (Gu et al., 2019), and lncRNA TUG1 can regulate the cigarette smoke induced airway remodeling by sponging miR-145-5p in COPD patients. Active nuclear factor kappa B (NF-κB) pathway is vital for the progression of the inflammatory response, and the protection of NF-κB inhibitor from inflammatory injury may be correlated with the lncRNA TUG1 (Cao et al., 2020). However, the biological role that lncRNA TUG1 plays in the hypersusceptibility of airway inflammatory response induced by respiratory viruses in COPD is barely reported.

The aim of this study is to investigate the underlying molecular mechanisms that the lncRNA TUG1/miR-145-5p/NF-κB pathway mediates in the hypersusceptibility of airway inflammatory response induced by H3N2 in COPD, thus exploring the underlying mechanisms why AECOPD patients induced by IAV have more severe symptoms and the possible therapeutic strategy.

## Materials and Methods

### Study Approval

The study was approved by the ethics committee of the First Affiliated Hospital of Anhui Medical University (Quick-PJ2019-15-22); all participants provided their written consent and were informed the purpose of this study. All experiments that involved working with the IAV H3N2 were performed according to the biosafety level two requirements and personal protection equipment was provided for all the researchers. The characteristics of all subjects were shown in Supplementary Table S1.

### Cell Isolation and Culture

pHBECs were isolated from the bronchial tissues of patients with lung carcinoma *in situ* comorbidity with and without COPD (DBHE and NHBE) according to the method modified from previous studies (Fulcher et al., 2005; Yamaya et al., 2011). The definition of COPD was based on the signs and symptoms and spirometry according to the 2017 GOLD guidelines (Vogelmeier et al., 2017). The bronchial tissues were cut at the site more than 2 cm distant from the edge of lung carcinoma. The isolated bronchial tissues were cut into pieces and incubated in solution comprised of 100 μg/ml pronase and 1 μg/ml deoxyribonuclease (both from Sigma-Aldrich) at 4°C overnight, then the supernatant was removed, and the tissue was resuspended and digested in another solution comprising of Ethylenediaminetetraacetic Acid (EDTA 2 nm), CaCl_2_ (0.75 mg/ml), MgCl_2_ (1 mg/ml), DL-Dithiothreitol (0.05 mg/ml), collagenase (0.25 mg/ml), and deoxyribonuclease (10 μg/ml) (all from Sigma-Aldrich) for 1 h. The pHBECs were finally isolated from the mixture with a centrifugation of 500 g 5 min and carefully rinsed with phosphate buffer solution (PBS) and plated on collagen-coated culture dishes (10 μg/cm^2^ rat type I collagen; Sigma) in bronchial epithelial growth medium (BEGM) (Lonza, NJ, United States), comprising bronchial epithelial basal medium (BEBM) supplemented with bovine pituitary extract (52 μg/ml), hydrocortisone (0.5 μg/ml), human recombinant epidermal growth factor (25 ng/ml), epinephrine (0.5 μg/ml), transferrin (10 μg/ml), insulin (5 μg/ml), retinoic acid (50 nm), triiodothyronine (6.5 ng/ml), gentamycin (40 μg/ml), amphotericin B (50 ng/ml), and bovine serum albumin (BSA) (1.5 μg/ml). The pHBECs were observed in the cultured dishes after 3–5 days, and the medium was replaced once every one or two days. After trypsinization (passage one), cells were cultured accordingly for different experiments, and all the experiments performed in this study with pHBECs were done on monolayer cultures. The identity of the monolayer as bronchial epithelial cells was confirmed as previously described (Ji et al., 2020).

### IAV Infection

pHBECs were seeded at a density of 1*10^6^ cells/well in 6-well plates coated with type I rat-tail collagen and cultured for 24 h to allow monolayer formation. Then cells were infected with IAV H3N2 (Multiplicity of Infection (MOI) = 2) for 24 h. The supernatants were collected and stored at −80°C for further examination; then the cells were rinsed with PBS three times and the protein or RNA was further extracted.

### Quantitative Reverse Transcriptase Polymerase Chain Reaction

RNA was extracted using TRIzol reagent kit (Invitrogen) and transcribed to cDNA using the high capacity cDNA reverse transcription. Quantitative reverse transcriptase polymerase chain reaction (qRT-PCR) was performed using the SYBR Premix Ex Taq II (Tli RNaseH Plus) (Takara Biotechnology, Dalian, China). The relative expression levels were calculated by the relative quantification (2^−△△Ct^) method, and data were normalized to β-actin as endogenous control. The following primer sequences were as follows:

TUG1 (human) forward: 5′-TGA​GCA​AGC​ACT​ACC​ACC​AG-3′, reverse: 5′-ACT​CAG​CAA​TCA​GGA​GGC​AC-3′; miR-145-5p (human) forward: 5′-CAG​TCT​TGT​CCA​GTT​TTC​CCA​G-3′, reverse: 5′-TAT​GCT​TGT​TCT​CGT​CTC​TGT​GTC-3′; NF-κBp65(human) forward: 5′-TGT​GAA​GAA​GCG​GGA​CCT​GGA​G-3′, reverse: 5′-AAG​CAG​AGC​CGC​ACA​GCA​TTC-3′; IL-1β (human) forward: 5′-CTC​CAC​CTC​CAG​GGA​CAG​GAT​ATG-3′, reverse:5′-TCATCTTTCAACACGCAGGACAGG-3′; TNF-α(human) forward:5′-TGGCGTGGAGCTGAGAGATAACC-3′, reverse: 5′-CGA​TGC​GGC​TGA​TGG​TGT​GG-3′; U6 (human) forward:5′-TGGAACGCTTCACGAATTTGCG-3′, reverse: 5′-AGA​CTG​CCG​CCT​GGT​AGT​TGT-3′

### Plasmid Construction, Lentivirus Production, and Cell Transduction

miR-145-5p mimic and its negative control mimic (miR-NC) and miR-145-5p inhibitor and its negative control inhibitor were purchased from Hanbio Biotechnology Co., Ltd. (Shanghai, China). The sequences were as follows:

miR-145-5p mimic:5′-GUCCAGUUUUCCCAGGAAUCCCU-3′ and 5′-AGG​GAU​UCC​UGG​GAA​AAC​UGG​AC-3′; negative control:5′-UCACAACCUCCUAGAAAGAGUAGA-3′ and 5′-UCU​ACU​CUU​UCU​AGG​AGG​UUG​UGA-3′; miR-145-5p inhibitor: 5′-AGG​GAU​UCC​UGG​GAA​AAC​UGG​AC-3′; scrambled control: 5′-UCU​ACU​CUU​UCU​AGG​AGG​UUG​UGA-3′.

The coding region of the NF-κBp65 mRNA was cloned into the pcDNA3.1 vector. The lentiviral vector expressing short hairpin RNA (shRNA) targeting TUG1 was designed and constructed by Hanbio Biotechnology Co., Ltd. (Shanghai, China). All cell transfection procedures were performed with Lipofectamine 3000 (Invitrogen, CA, United States) according to the manufacturer’s instructions.

### Western Blotting Analysis

Total protein extracts were prepared by using RIPA lysis buffer (ShangHai Biocolor BioScience Technology Company, Shanghai, China) with phosphatase and protease inhibitors. Nuclear proteins were extracted with the nuclear and cytoplasmic extraction kit (ShangHai Beyotime Biotechnology Company, Shanghai, China), and the efficacy of the fractionation was evaluated by the expression of nuclear protein Histone H3 and cytoplasmic protein β-actin in nuclear protein with Western blotting. The protein concentration was determined by BCA assay (Thermo Scientific, Rockford, IL, United states). Equal amounts of protein extracts were separated by SDS-PAGE (Bio-Rad, Hercules, CA, United States) and electrophoretically transferred to a PVDF membrane (Millipore, Bedford, NY, United States). After blockade with BSA for 2 h at room temperature, membrane was incubated with human primary antibodies specific to phospho-NF-κBp65(Ser536) (1:1,000, Cell Signaling Technology, MA, United States), NF-κBp65 (1:1,000, Cell Signaling Technology, MA, United States), Histone H3 (1:1,000, Cell Signaling Technology, MA, United States), and β-actin (1:1,000, Cell Signaling Technology, MA, United States) at 4°C overnight. And subsequent incubation was done with a secondary horseradish peroxidase- (HRP-) conjugated antibody (KPL, Gaithersburg, MD, United States) at 1:5,000 dilution for 2 h at room temperature. The specific bands were visualized using the ECL detection kit (Thermo Fisher ScientificTM, Cleveland, OH, United States). Protein bands intensities were analyzed by Quantity One software (Bio-Rad Laboratories, Hercules, CA, United States).

### Cell Immunofluorescence

pHBECs were grown on 15 mm Glass Bottom Cell Culture Dish (NEST, China) and then infected with IAV H3N2 (MOI = 2); cells were fixed at different time points (0, 2, 4, and 8 h post infection (hpi)) for 10 min with 4% phosphate-buffered paraformaldehyde at room temperature. Then the fixing solution was aspirated off and cells were rinsed with PBS and permeabilized with 0.5% Trixton-100 for 10 min. The cells were then rinsed with PBS and blocked with 5% BSA (FractionV, Beyotime, China) for 1 h. After incubation with the primary antibody of NF-κBp65 (1:200, Cell Signaling Technology, Beverly, MA, United States) at 4°C overnight, cells were rinsed for three times with PBS and incubated with the secondary antibody conjugated with FITC at a 1:500 dilution for 1 h at room temperature; the cells were followed by three rinsing steps for 5 min with PBS. Cells nuclei were stained using 4, 6-diamidino-2-pheylindole (DAPI, Sigma) and analyzed using a confocal laser scanning microscope (Leica SP8).

### ELISA

Cell culture supernatants were collected and frozen at −80°C. The protein expression of proinflammatory cytokines (TNF-α and IL-1β) was quantified by ELISA kits purchased from Dakewe Biological Technology Co., Ltd. (Beijing, China), according to the manufacturer’s instructions.

### Luciferase Reporter Assay

Plasmid pmirGLO-TUG1 wildtype (wt) or pmirGLO-TUG1 mutant (mut) (relevant binding sites in miR-145-5p) was cotransfected with miR-145-5p mimics or miR-NC into HEK293T cells using a Lipofectamine 3000-mediated gene transfer. NF-κBp65-wt/mut 3UTR was constructed and transfected into HEK293T cells along with miR-145-5p mimic/miR-NC. Luciferase activity was detected using the Dual-Luciferase Reporter Assay System (Promega, Madison, WI, United States) according to the manufacturer’s instructions.

### Statistical Analysis

Data were expressed as the mean ± SD. Statistical analysis was performed using Prism software (version 6, GraphPad Software, La Jolla, CA, United States); differences between groups were analyzed by one-way analysis of variance (ANOVA), followed by the Student-Newman-Keuls test. Statistically significant differences were accepted at *p* < 0.05.

## Results

### IAV Activates the NF-κB Pathway in pHBECs

**FIGURE 1:**
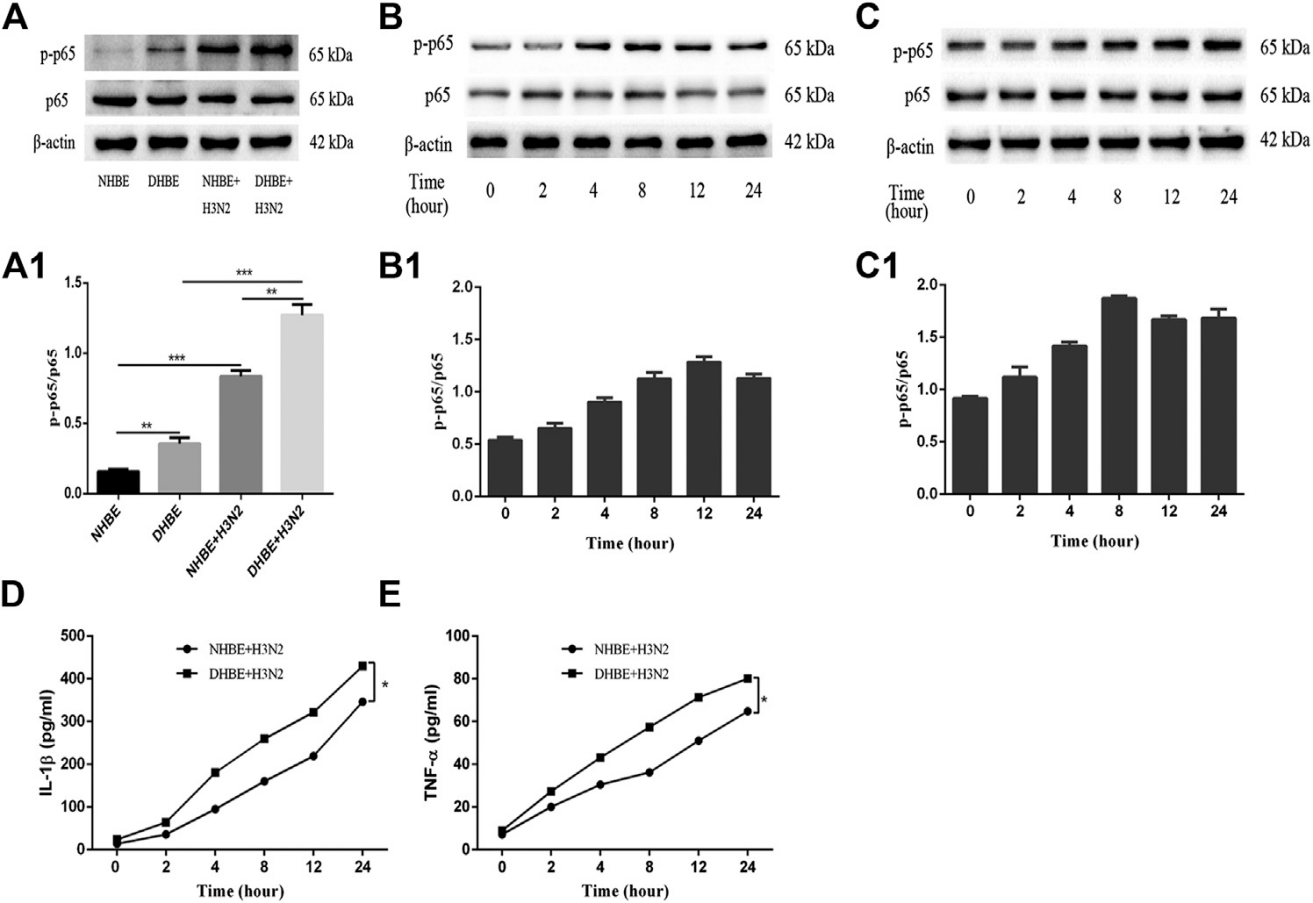
IAV activated the NF-κB pathway in pHBECs. **(A,A1)** The protein expression and relative intensity of NF-κBp65 and phospho-p65 in pHBECs after infection with or without IAV for 24 h. **(B,B1)** The time-dependent protein expression and relative intensity of NF-κBp65 and phospho-p65 in NHBE after infection with IAV. **(C,C1)** The time-dependent protein expression and relative intensity of NF-κBp65 and phospho-p65 in DHBE after infection with IAV. **(D,E)** ELISA analyses of IL-1β and TNF-α in pHBECs after infection with IAV (^*^ *p*<0.05). Each dataset comprises three independent experiments.

**FIGURE 2:**
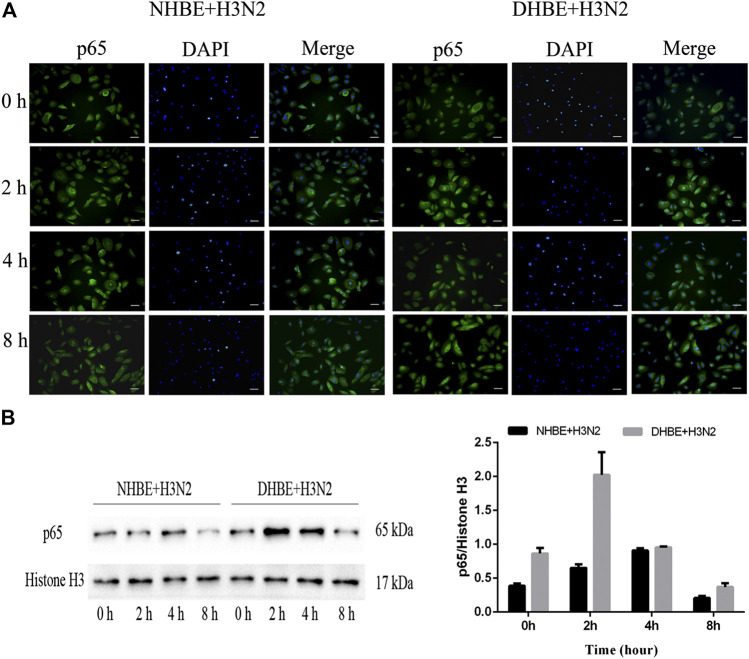
IAV activated the NF-κB pathway earlier in DHBE. **(A)** Cell immunofluorescence of IAV induced NF-κBp65 nuclear translocation in pHBECs measured at different times. The IAV induced NF-κBp65 nuclear translocation was obviously detected at four hpi in NHBE **(left)** and two hpi in DHBE **(right)**, scale bar = 50 μm. Note: hpi: hours post infection, **(B)** NF-κB/p65 expression and relative intensity in the nucleus of pHBECs after infection with IAV for different times.

The activity of NF-κB pathway was measured through the levels of NF-κBp65 and phospho-NF-κBp65. The expression of phospho-NF-κBp65 in DHBE was significantly higher than that in NHBE (*p*<0.01); after infection with IAV for 24 h, the expression of phospho-NF-κBp65 increased significantly in pHBECs (*p*<0.001) (shown in Figures 1A, A1). The detailed time analysis revealed that the expression of phospho-NF-κBp65 in pHBECs changed in a time-dependent manner (shown in Figures 1B, B1, C, C1); moreover, the intensity analysis showed the peak expression of phospho-NF-κBp65 in DHBE was observed at 8 hpi, earlier than that at 12 hpi in NHBE. Meanwhile, the IAV induced NF-κBp65 nuclear translocation was measured by immunofluorescence; as shown in Figure 2A, the IAV induced NF-κBp65 nuclear translocation in DHBE was obviously detected at two hpi and sustained up to four hpi, while in NHBE, the NF-κBp65 nuclear translocation was barely detected at two hpi and apparently increased at four hpi. The corresponding expressions of NF-κBp65 in the nucleus were also detected by Western blotting, as shown in Figure 2B. The efficacy of nuclear fractionation in nuclear protein was evaluated by Western blotting, as shown in Supplementary Figure S1. All these results may indicate that the NF-κB pathway in DHBE was activated earlier than NHBE after infection with IAV.

The downstream proinflammatory cytokines (IL-1β and TNF-α) of NF-κB pathway were quantified by ELISA. As shown in Figures 1D, E, IAV infection increased the expressions of IL-1β and TNF-α significantly in a time-dependent manner (*p*<0.05). Moreover, the expressions of IL-1β and TNF-α measured in the culture supernatants of DHBE were significantly higher than those of NHBE at different time points.

### The Differential Expressions of lncRNA TUG1 and miR-145-5p in COPD

**FIGURE 3:**
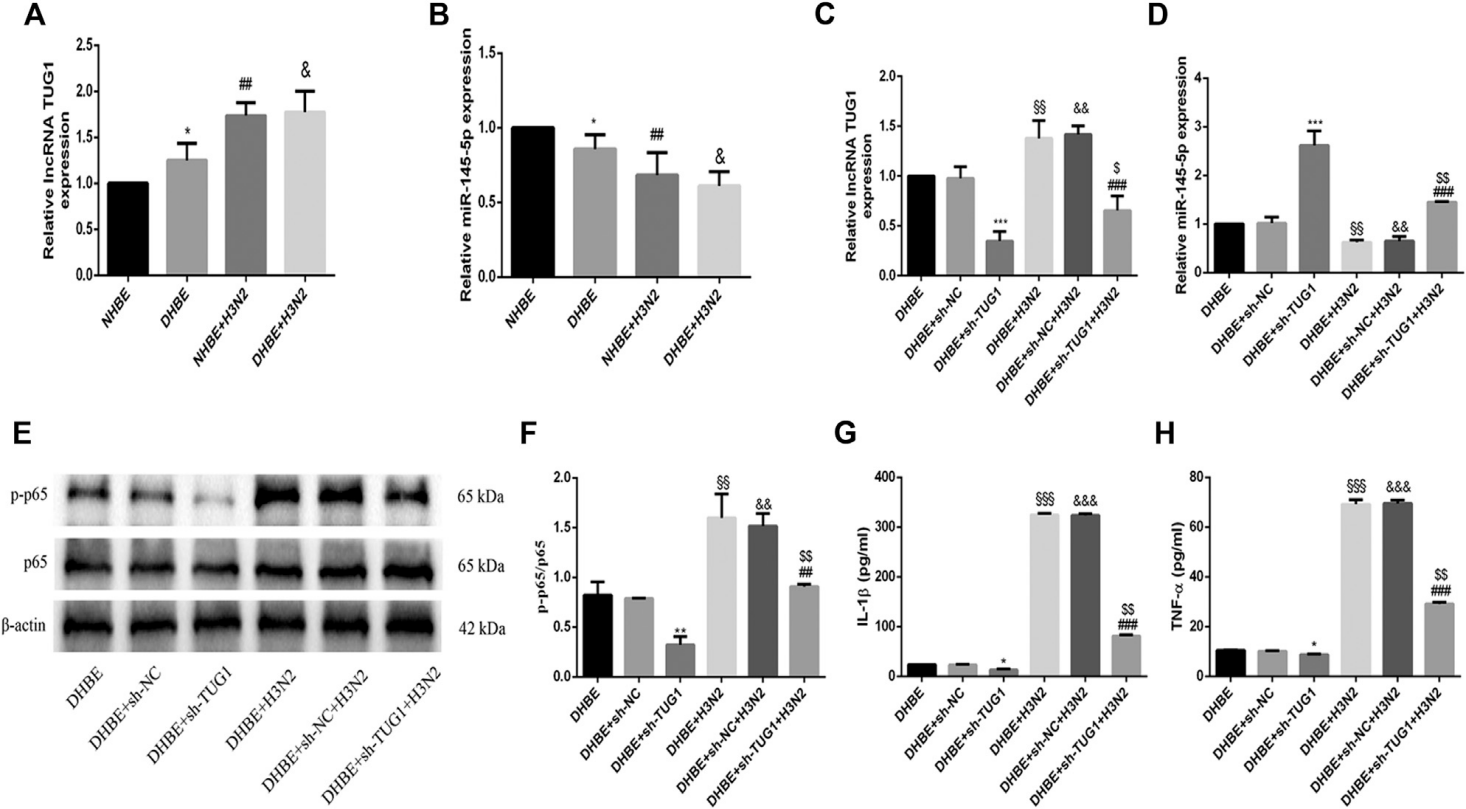
Knockdown of lncRNA TUG1 reduced the IAV induced airway inflammation in DHBE. **(A,B)** The expression of lncRNA TUG1 and miR-145-5p was analyzed in pHBECs with or without IAV infection for 24 h (note: ^*^ *p*<0.05 as compared to NHBE, ^##^ *p*<0.01 as compared to NHBE, ^&^ *p*<0.05 as compared to DHBE). **(C,D)** The mRNA levels of lncRNA TUG1 and miR-145-5p were analyzed in DHBE pretreated with or without sh-TUG1. **(E,F)** The protein expressions of NF**-**κBp65 and phospho-NF**-**κBp65 were analyzed in DHBE pretreated with or without sh-TUG1 by Western blotting. **(G,H)** The protein expressions of IL-1β and TNF-α were analyzed in DHBE pretreated with or without sh-TUG1 by ELISA. (^*^ *p*<0.05, ^**^ *p*<0.01, ^***^ *p*<0.001 as compared to DHBE + sh-NC group, ^##^ *p*<0.01, ^###^ *p*<0.001 as compared to DHBE + H3N2 group, ^§§^ *p*<0.01, ^§§§^ *p*<0.001 as compared to DHBE group, ^&&^ *p*<0.01, ^&&&^ *p*<0.001 as compared to DHBE + sh-NC group, ^$^ *p*<0.05, ^$$^ *p*<0.01 as compared to DHBE + sh-NC + H3N2 group). Each dataset comprises three independent experiments.

The relative expressions of lncRNA TUG1 and miR-145-5p in pHBECs were examined with qRT-PCR. As shown in Figure 3A, the expression of lncRNA TUG1 measured in DHBE was significantly higher than that in NHBE (*p*<0.05), but opposite in the expression of miR-145-5p (Figure 3B, *p*<0.05). After infection with the IAV for 24 h, the expression of lncRNA TUG1 in pHBECs increased significantly, whereas the expression of miR-145-5p decreased significantly.

### Knockdown of lncRNA TUG1 Reduced Airway Inflammation Induced by IAV in DHBE

To investigate whether the IAV induced airway inflammation was regulated by the lncRNA TUG1, we pretreated the DHBE with shRNA specific to lncRNA TUG1 (sh-TUG1). As shown in Figure 3C, the expression of lncRNA TUG1 in DHBE attenuated significantly after treatment with sh-TUG1. Although the mRNA levels of NF-κBp65, IL-1β, and TNF-α in DHBE were increased significantly after infection with IAV, this effect was reduced significantly by sh-TUG1 (Supplementary Figure S2); consistent with this result, the protein expressions of phospho-NF-κBp65, IL-1β, and TNF-α were also reduced significantly by sh-TUG1 (shown in Figures 3E–H). These results indicated that knockdown of lncRNA TUG1 reduced airway inflammation induced by the IAV in DHBE.

### lncRNA TUG1 Positively Regulated the IAV Induced Airway Inflammation by Sponging miR-145-5p in DHBE

**FIGURE 4:**
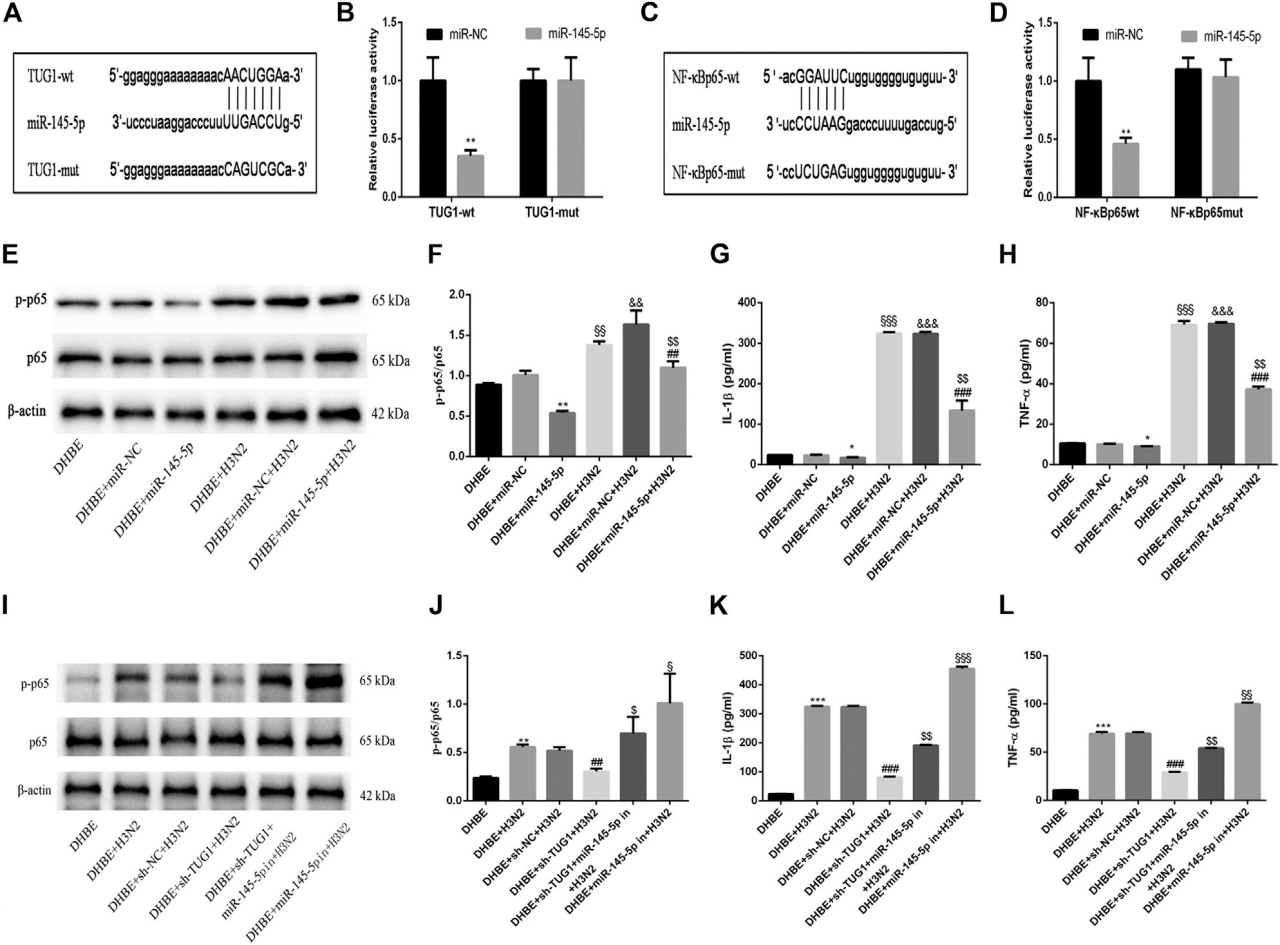
lncRNA TUG1 positively regulated the IAV induced airway inflammation by sponging miR-145-5p in DHBE. **(A)** The putative miR-145-5p binding sequence of TUG1 mRNA. **(B)** Luciferase activity was measured between miR-145-5p (wt and mut) and TUG1 (wt and mut). **(C)** The putative miR-145-5p binding sequence of the 3′UTR of NF-κBp65. **(D)** Luciferase activity was measured between miR-145-5p (wt and mut) and NF-κBp65 (wt and mut). **(E,F)** The protein expressions of NF**-**κBp65 and phospho-NF**-**κBp65 were analyzed in DHBE pretreated with or without miR-145-5p mimic by Western blotting. **(G,H)** The protein expressions of proinflammatory cytokines IL-1β and TNF-α were analyzed in DHBE pretreated with or without miR-145-5p mimic by ELISA (^*^ *p*<0.05, ^**^ *p*<0.01 as compared to DHBE + miR-NC group, ^##^ *p*<0.01, ^###^ *p*<0.001 as compared to DHBE + H3N2 group,^§§^ *p*<0.01, ^§§§^ *p*<0.001 as compared to DHBE group, ^&&^ *p*<0.01, ^&&&^ *p*<0.001 as compared to DHBE+ miR-NC group, ^$$^ *p*<0.01 as compared to DHBE+ miR-NC +H3N2 group). Each dataset comprises three independent experiments. **(I,J)** The protein expressions of NF**-**κBp65 and phospho-NF**-**κBp65 were analyzed in DHBE pretreated with or without sh-TUG1/miR-145-5p inhibitor by Western blotting. **(K,L)** The protein expressions of proinflammatory cytokines IL-1β and TNF-α were analyzed in DHBE pretreated with or without sh-TUG1/miR-145-5p inhibitor by ELISA (^**^ *p*<0.01, ^***^ *p*<0.001 as compared to DHBE group, ^##^ *p*<0.01, ^###^ *p*<0.001 as compared to DHBE + sh-NC + H3N2 group, ^$^ *p*<0.05, ^$$^ *p*<0.01 as compared to DHBE+ sh-TUG1+H3N2 group, ^§^ *p*<0.05, ^§§^ *p*<0.01, ^§§§^ *p*<0.001 as compared to DHBE+ sh-TUG1+miR-145-5p in+H3N2 group). Each dataset comprises three independent experiments.

To investigate the mechanisms by which the lncRNA TUG1 regulates the IAV induced airway inflammation, we predicted the miRNA target sites with bioinformatic database analysis and identified the miR-145-5p as the lncRNA with relevant binding site in the *TUG1* mRNA. We then constructed luciferase reporter vectors that contained the wildtype (wt) or mutated (mut) binding sequences for miR-145-5p in TUG1, and luciferase reporter assay results displayed that luciferase activity was suppressed in TUG1-wt cells but was not in TUG1-mut cells (Figures 4A, B), which suggested that miR-145-5p is a TUG1-targeting miRNA. Moreover, we demonstrated that miR-145-5p expression was significantly upregulated by lncRNA TUG1 knockdown in DHBE (Figure 3D). Bioinformatic database analysis revealed that miR-145-5p interacted with the 3UTR of NF-κBp65 mRNA. Dual-luciferase reporter assay revealed that the luciferase activity was significantly suppressed when cotransfected with NF-κBp65-wt and miR-145-5p mimic, whereas cotransfection with NF-κBp65-mut and miR-145-5p mimic failed to affect the luciferase activity (Figures 4C, D). The pretransfection of miR-145-5p mimic in DHBE partially inhibited the activation of NF-κB pathway induced by IAV infection, as well as the expressions of IL-1β and TNF-α (shown in Figures 4E–H and Supplementary Figure S3).

In addition, the lncRNA TUG1 knockdown attenuated the expressions of phospho-NF-κBp65, IL-1β and TNF-α significantly in DHBE after infection with IAV, and this effect was reversed by miR-145-5p inhibition, as shown in Figures 4I–L and Supplementary Figure S4. These results suggested that the lncRNA TUG1 positively regulated the IAV induced airway inflammation by sponging miR-145-5p in DHBE.

### Overexpression of NF-κBp65 Reversed the Effects of lncRNA TUG1 Knockdown in DHBE After Infection With IAV

**FIGURE 5:**
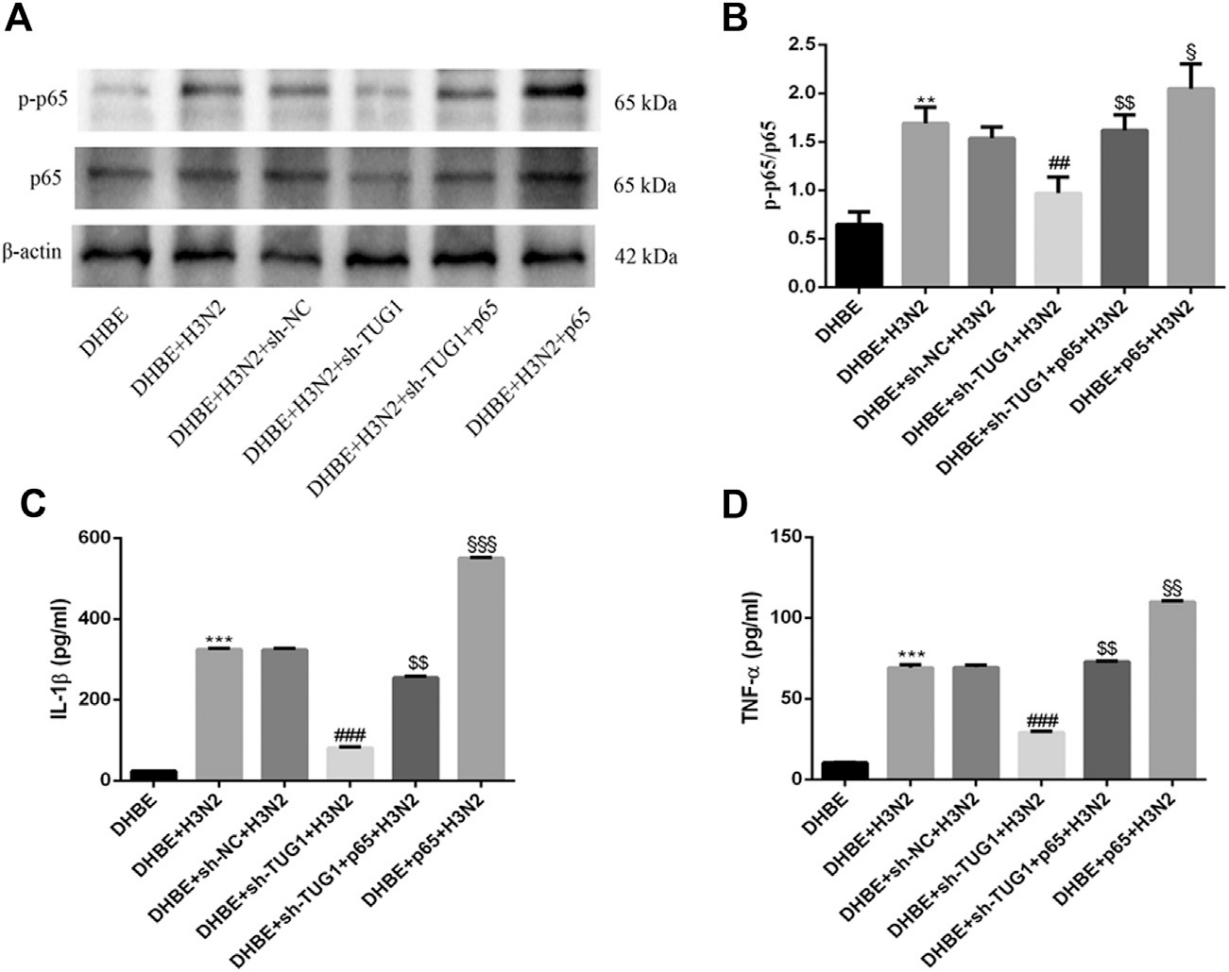
Overexpression of NF-κBp65 reversed the effects of lncRNA TUG1 knockdown in DHBE after infection with IAV. **(A,B)** The protein expressions of NF**-**κBp65 and phospho-NF**-**κBp65 were analyzed in DHBE pretreated with or without sh-TUG1 and NF-κBp65 plasmid by Western blotting. **(C,D)** The protein expressions of proinflammatory cytokines IL-1β and TNF-α were analyzed in DHBE pretreated with or without sh-TUG1 and NF-κBp65 plasmid by ELISA (^**^ *p*<0.01, ^***^ *p*<0.001 as compared to DHBE group, ^##^ *p*<0.01, ^###^ *p*<0.001 as compared to DHBE + sh-NC + H3N2 group, ^$$^ *p*<0.01 as compared to DHBE + sh-TUG1+H3N2 group, ^§^ *p*<0.05, ^§§^ *p*<0.01, ^§§§^ *p*<0.001 as compared to DHBE + sh-TUG1+p65 + H3N2 group). Each dataset comprises three independent experiments.

**FIGURE 6:**
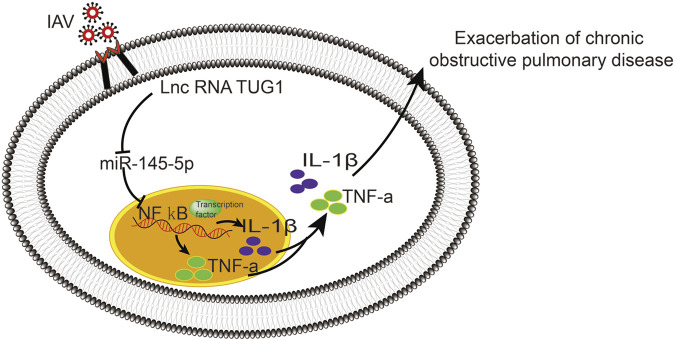
Schematic diagram to illustrate the interactions of lncRNA TUG1/miR-145-5p/NF-κB pathway.

To further investigate the interaction of lncRNA TUG1 and NF-κB pathway, we performed gain-of-function assays by introducing the NF-κBp65 overexpression plasmid into DHBE with lncRNA TUG1 knockdown. As shown in Figures 5A, B and Supplementary Figure S5A, lncRNA TUG1 knockdown attenuated phospho-NF-κBp65 expression in IAV infected DHBE, whereas NF-κBp65 overexpression reversed this effect. Furthermore, NF-κBp65 overexpression attenuated the suppressive effect of lncRNA TUG1 knockdown on the expressions of IL-1β and TNF-α in IAV infected DHBE (shown in Figures 5C, D and Supplementary Figures S5B, C). These results revealed that lncRNA TUG1 knockdown might have a protective effect on the airway inflammatory response by repressing the activation of NF-κB pathway. We provide a schematic diagram to better illustrate the interactions among lncRNA TUG1/miR-145-5p/NF-κB pathway, as shown in Figure 6.

## Discussion

COPD patients are more susceptible to influenza virus infection, but the underlying molecular mechanism of its higher susceptibility is unknown. Respiratory epithelial cells are the first physical barrier to defense against respiratory viruses; they rapidly recognize the virus antigen with a variety of pattern recognition receptors, trigger the inflammatory responses by releasing cytokines, and regulate potentially harmful inflammation (Sajjan, 2013). In the present study, we demonstrated an earlier activation of NF-κB pathway in DHBE after IAV infection, accompanied with increased lncRNA TUG1 and decreased miR-145-5p. Knockdown of lncRNA TUG1 significantly attenuated the airway inflammation by inhibiting the NF-κB pathway and its downstream proinflammatory cytokines IL-1β and TNF-α. Meanwhile, mechanistic analysis revealed that the lncRNA TUG1 positively regulates the NF-κB pathway by sponging miR-145-5p.

NF-κB pathway is a key mediator in inflammatory injury and has been proven to participate in various cellular signaling transduction, including the influenza virus infection. Nimmerjahn and colleagues (Nimmerjahn et al., 2004) showed that an active NF-κB pathway was a prerequisite for influenza virus infection. Although previous research has studied the IAV induced inflammatory response in pHBECs (Hsu et al., 2017; Huo et al., 2018), little have investigated the difference of the hypersusceptibility of airway inflammatory response caused by IAV between normal and COPD patients. Here we not only confirmed the activated NF-κB pathway in pHBECs after IAV infection, but also compared the differences in activation of NF-κB pathway between NHBE and DHBE at different time points. We found that the peak expression of phospho-NF-κBp65 observed in DHBE was 4 h earlier than NHBE with Western blotting, and the IAV induced NF-κBp65 nuclear translocation in DHBE was 2 h earlier than NHBE with cell immunofluorescence. The individual variation in susceptibility to virus directly affects the inflammatory responses of people with different immune status. An active NF-κB pathway may directly induce the exaggerated “cytokine storm” in the early stage after IAV infection (Nimmerjahn et al., 2004), and the inflammatory cytokines release may be quicker and stronger in COPD patients. Our results may partially explain the reason why COPD patients are more susceptible to IAV and have earlier and more severe symptoms than normal people, as well as the higher morbidity and mortality of emerging infectious diseases like Severe Acute Respiratory Syndrome (SARS) (Xu et al., 2020). All these indicate that we need to pay more attention and implement effective strategies to prevent the inflammatory injuries in the early stage of diseases.

lncRNA regulates the gene expression at the various levels, including transcriptional and posttranscriptional ones. Aberrant lncRNA may lead to dysfunction and contribute to the pathogenesis of various diseases (Chen, 2016). It interacts with a specific protein and regulates its functions or activities; moreover, lncRNA may interfere with the transcription by regulating the encoding sequences of protein genes (Shi et al., 2013). Furthermore, lncRNA can modulate a variety of biological activities or functions by acting as a competing endogenous RNA or miRNA sponge (Gu et al., 2019). In this study, we predicted the miR-145-5p as the binding target of lncRNA TUG1 with bioinformatic database analysis. We found that the DHBE had a significantly higher lncRNA TUG1 expression and lower miR-145-5p expression than NHBE, and the lncRNA TUG1 could regulate the airway inflammatory response by sponging miR-145-5p, which was consistent with Gu’s study (Gu et al., 2019). Zhang et al. (Zhang et al., 2020) analyzed the expression profiles of lncRNAs and mRNAs in H3N2 infection by RNA sequencing. Different from our study, they did not report the expression of lncRNA TUG1 or miR-145-5p; we speculate this difference may be explained by the different research object we studied, and the pHBECs we studied may be more similar to the result *in vivo*. Previous studies have shown that the miR-145-5p attenuated significantly after being exposed to cigarette smoke extract and negatively regulated the proinflammatory cytokines expressions of airway smooth muscle cells in COPD patients (Pal et al., 2013; O'Leary et al., 2016). Here we found that the miR-145-5p expression in the pHBECs decreased significantly after IAV infection, accompanied with increased lncRNA TUG1 expression, activated NF-κB pathway, and increased IL-1β and TNF-α. Furthermore, this effect was significantly reversed by downregulation of lncRNA TUG1 or miR-145-5p mimic, which was quite consistent with Wang’s study (Wang et al., 2019b) that the overexpression of miR-145-5p can inhibit the secretion of proinflammatory cytokines by suppressing NF-κB pathway in high glucose-induced retinal endothelial cells. It is reasonable to speculate that the NF-κB pathway was regulated positively by lncRNA TUG1 and negatively by miR-145-5p.

## Conclusion

In conclusion, the present study demonstrated that the IAV can induce an exaggerated hypersusceptibility of airway inflammatory response in COPD by activating the lncRNA TUG1/miR-145-5p/NF-κB pathway *in vitro*, which may associate with the more severe symptoms induced by IAV in COPD. We speculate that the lncRNA TUG1 inhibitor may be a promising therapeutic strategy for AECOPD caused by IAV.

## Data Availability

The original contributions presented in the study are included in the article/Supplementary Material; further inquiries can be directed to the corresponding author.
