# Supplementary figures and images for: The Influenza A Virus H3N2 Triggers the Hypersusceptibility of Airway Inflammatory Response via Activating the lncRNA TUG1/miR-145-5p/NF-κB Pathway in COPD
Source: Front Pharmacol. 2021 Feb 22;12:604590. doi: 10.3389/fphar.2021.604590 (PMC8029562; doi:10.3389/fphar.2021.604590)

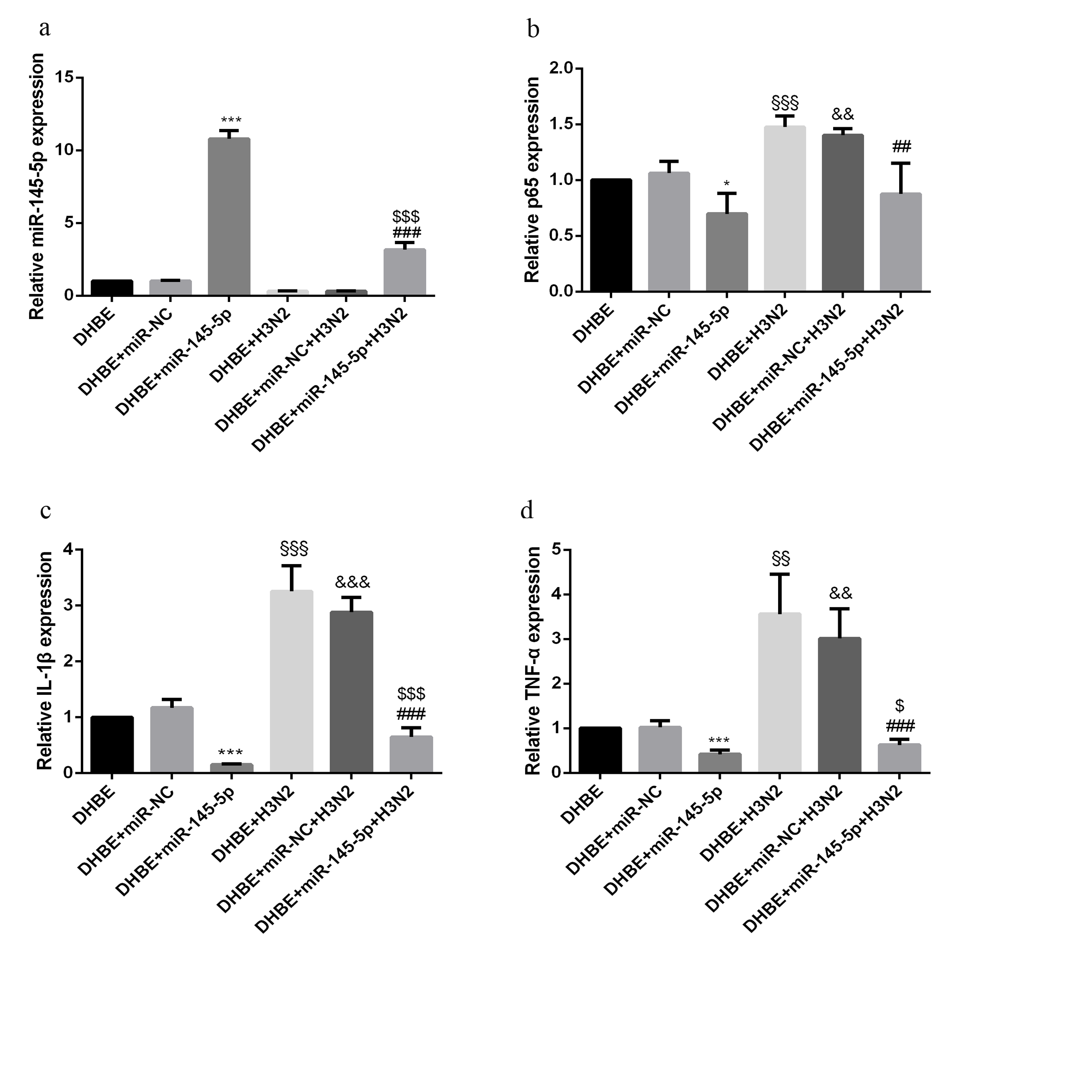

Supplement: Supplementary file 1 [file image3.tif]

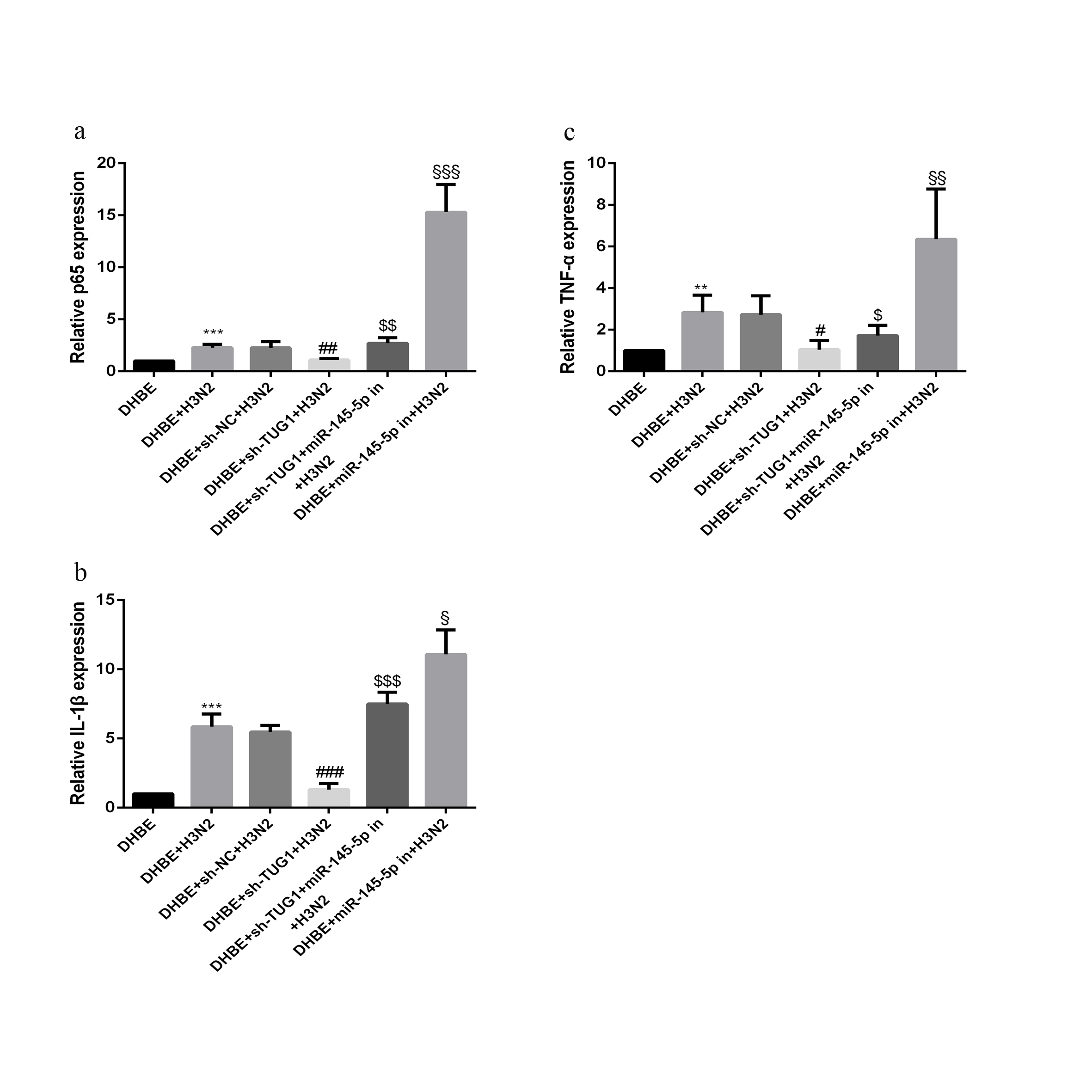

Supplement: Supplementary file 2 [file image4.tif]

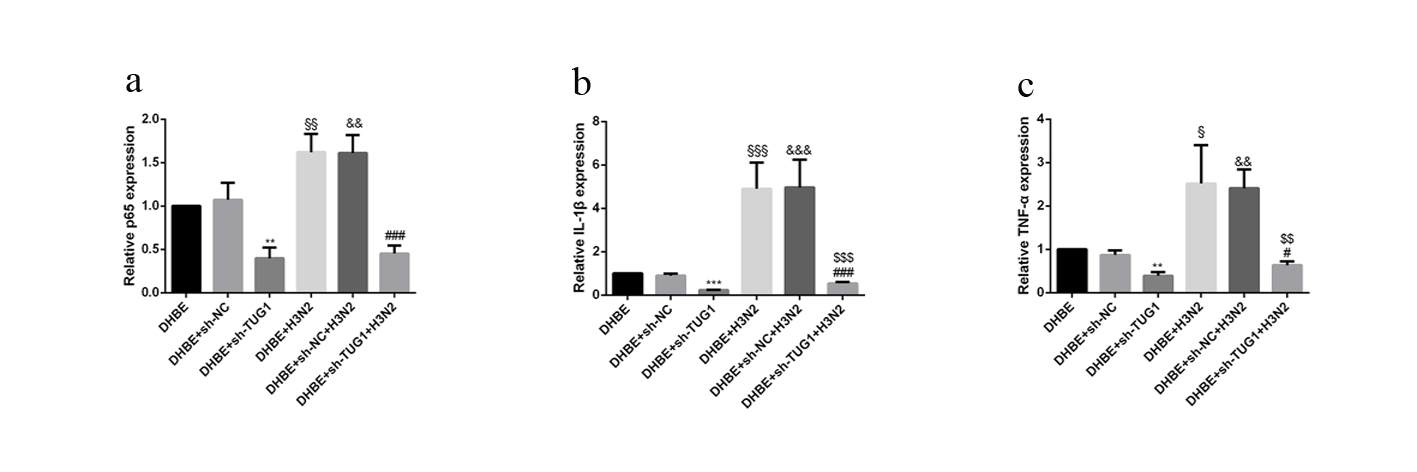

Supplement: Supplementary file 3 [file image2.tif]

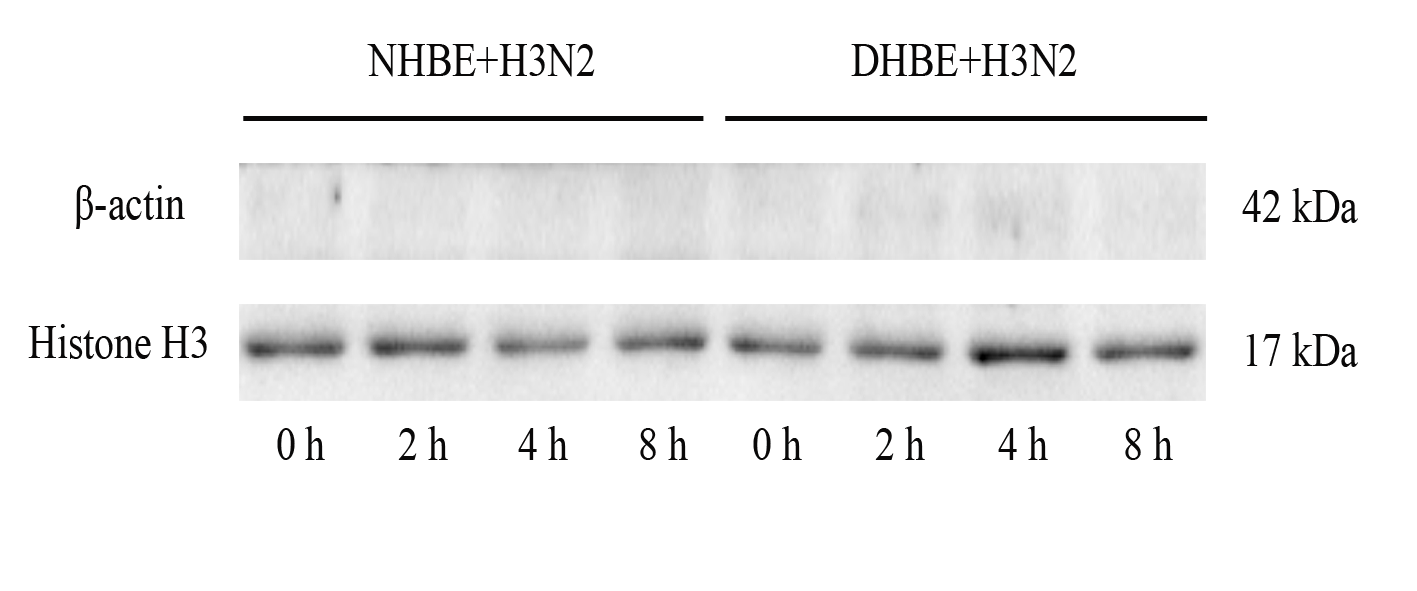

Supplement: Supplementary file 4 [file image1.tif]

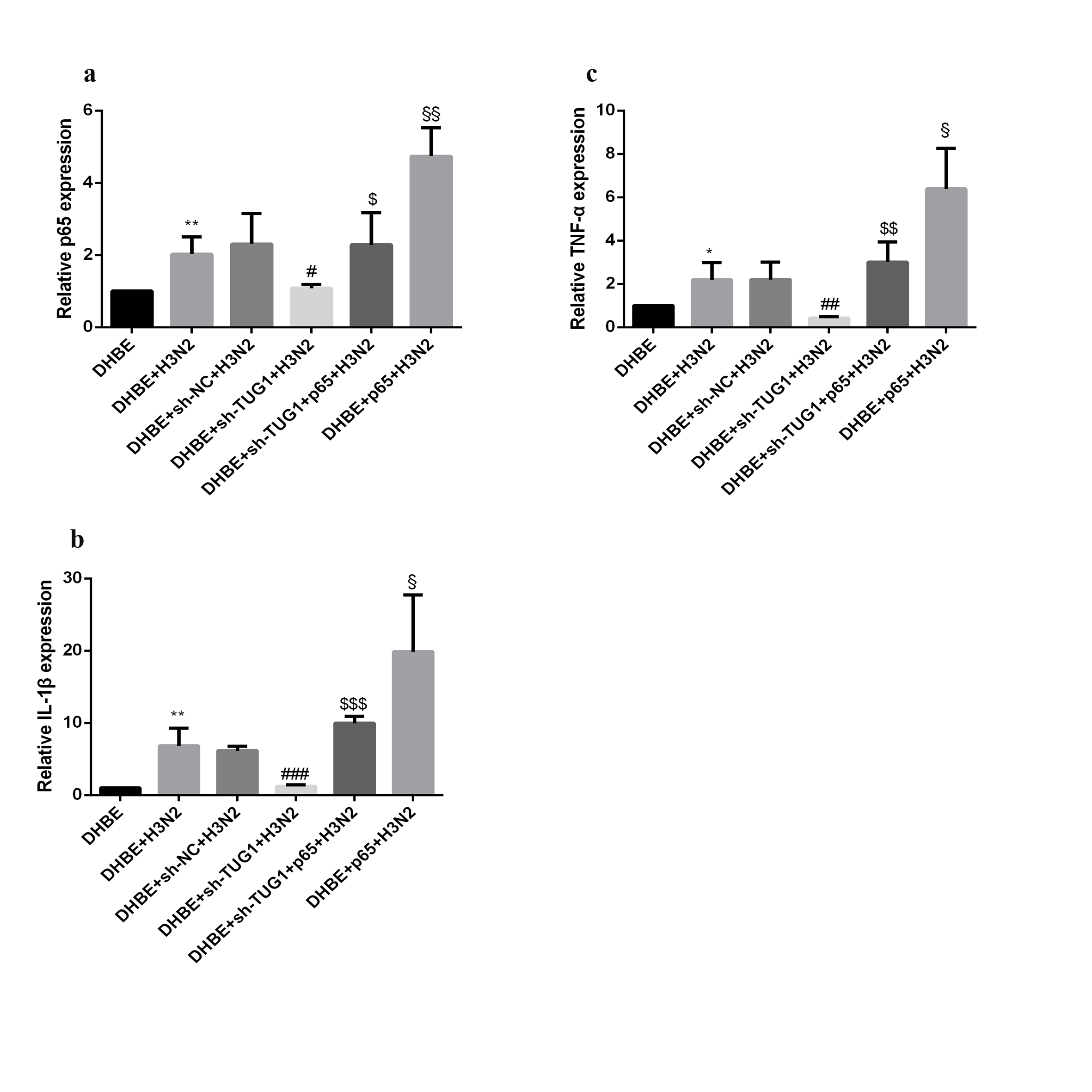

Supplement: Supplementary file 5 [file image5.tif]
