# Supplementary material for: The Influenza A Virus H3N2 Triggers the Hypersusceptibility of Airway Inflammatory Response via Activating the lncRNA TUG1/miR-145-5p/NF-κB Pathway in COPD
Source: Front Pharmacol. 2021 Feb 22;12:604590. doi: 10.3389/fphar.2021.604590 (PMC8029562; doi:10.3389/fphar.2021.604590)
Supplement: Supplementary file 6 [file datasheet1.docx]

**Supplemental material**

Table 1. Characteristics of all subjects

| Characteristic | Normal group  (n=6) | COPD group  (n=5) | *P* value |
| --- | --- | --- | --- |
| Age ( years)  BMI (kg/m^2^)  Sex(F/M)  Smoking index  FEV_1_%  FEV_1_/FVC(%) | 66.56±10.34  24.23±2.98  3/3  6.78±9.56  100.16±20.82  80.78±5.91 | 65.23±11.56  23.95±3.56  2/3  17.96±15.28  45.68±10.57  40.95±6.96 | 0.28  0.38  0.61  0.000  0.000  0.000 |

Note: Data are presented as means±SD. *P* values were calculated by independent t test. BMI: body mass index. FEV1%: predicted forced expiratory volume in 1second. FEV1/FVC: ratio of forced expiratory volume in 1second to forced vital capacity.

**Supplemental Figure 1**

The fractionation in nuclear proteins of pHBECs after infection with IAV for different times was evaluated by western blotting.

**Supplemental Figure 2**

The mRNA levels of NF**-**κB/p65 (a)，IL-1β (b) and TNF-α (c) were analysed in DHBE pre-treated with or without sh-TUG1 by qRT-PCR.

(^**^*P*＜0.01, ^***^*P*＜0.001 as compared to DHBE+sh-NC group, ^#^*P*＜0.05, ^###^*P*＜0.001 as compared to DHBE+H3N2 group, ^§^*P*＜0.05, ^§§^*P*＜0.01, ^§§§^*P*＜0.001 as compared to DHBE group, ^&&^*P*＜0.01, ^&&&^*P*＜0.001 as compared to DHBE+sh-NC group, ^$$^*P*＜0.01, ^$$$^*P*＜0.001 as compared to DHBE+sh-NC+H3N2 group). Each dataset comprises three independent experiments.

**Supplemental Figure 3**

The mRNA levels of miR-145-5p (a), NF**-**κB/p65 (b)，IL-1β (c) and TNF-α (d) were analysed in DHBE pre-treated with or without miR-145-5p mimic by qRT-PCR.

(^*^*P*＜0.05, ^***^*P*＜0.001 as compared to DHBE+miR-NC group, ^##^*P*＜0.01, ^###^*P*＜0.001 as compared to DHBE+H3N2 group,^§§^*P*＜0.01, ^§§§^*P*＜0.001 as compared to DHBE group, ^&&^*P*＜0.01, ^&&&^*P*＜0.001 as compared to DHBE+miR-NC group, ^$^*P*＜0.05, ^$$$^*P*＜0.001 as compared to DHBE+miR-NC+H3N2 group). Each dataset comprises three independent experiments

**Supplemental Figure 4**

The mRNA levels of NF**-**κB/p65 (a)，IL-1β (b) and TNF-α (c) were analysed in DHBE pre-treated with or without miR-145-5p inhibitor by qRT-PCR.

(^**^*P*＜0.01, ^***^*P*＜0.001 as compared to DHBE group, ^#^*P*＜0.05, ^##^*P*＜0.01, ^###^*P*＜0.001 as compared to DHBE+sh-NC+H3N2 group, ^$^*P*＜0.05, ^$$^*P*＜0.01, ^$$$^*P*＜0.001 as compared to DHBE+ sh-TUG1+H3N2 group, ^§^*P*＜0.05, ^§§^*P*＜0.01, ^§§§^*P*＜0.001 as compared to DHBE+sh-TUG1+miR-145-5p in+H3N2 group). Each dataset comprises three independent experiments.

**Supplemental Figure 5**

The mRNA levels of NF**-**κB/p65 (a)，IL-1β (b) and TNF-α (c) were analysed in DHBE pre-treated with or without sh-TUG1 and NF-κBp65 plasmid by qRT-PCR.

(^*^*P*＜0.05, ^**^*P*＜0.01 as compared to DHBE group, ^#^*P*＜0.05, ^##^*P*＜0.01, ^###^*P*＜0.001 as compared to DHBE+sh-NC+H3N2 group, ^$^*P*＜0.05, ^$$^*P*＜0.01, ^$$$^*P*＜0.001 as compared to DHBE+ sh-TUG1+H3N2 group, ^§^*P*＜0.05, ^§§^*P*＜0.01 as compared to DHBE+sh-TUG1+p65+H3N2 group). Each dataset comprises three independent experiments.
